# Survey of drug resistance associated gene mutations in *Mycobacterium tuberculosis*, ESKAPE and other bacterial species

**DOI:** 10.1038/s41598-020-65766-8

**Published:** 2020-06-02

**Authors:** Abhirupa Ghosh, Saran N., Sudipto Saha

**Affiliations:** Division of Bioinformatics, Bose Institute, Kolkata, India

**Keywords:** Genetic databases, Antimicrobial resistance

## Abstract

Tuberculosis treatment includes broad-spectrum antibiotics such as rifampicin, streptomycin and fluoroquinolones, which are also used against other pathogenic bacteria. We developed Drug Resistance Associated Genes database (DRAGdb), a manually curated repository of mutational data of drug resistance associated genes (DRAGs) across ESKAPE (i.e. *Enterococcus faecium*, *Staphylococcus aureus*, *Klebsiella pneumonia*e, *Acinetobacter baumannii*, *Pseudomonas aeruginosa*, and *Enterobacter* spp.) pathogens, and other bacteria with a special focus on *Mycobacterium tuberculosis* (MTB). Analysis of mutations in drug-resistant genes listed in DRAGdb suggested both homoplasy and pleiotropy to be associated with resistance. Homoplasy was observed in six genes namely *gidB*, *gyrA*, *gyrB*, *rpoB*, *rpsL* and *rrs*. For these genes, drug resistance-associated mutations at codon level were conserved in MTB, ESKAPE and many other bacteria. Pleiotropy was exemplified by a single nucleotide mutation that was associated with resistance to amikacin, gentamycin, rifampicin and vancomycin in *Staphylococcus aureus*. DRAGdb data also revealed that mutations in some genes such as pncA, *inhA*, *katG* and *embA,B,C* were specific to *Mycobacterium species*. For *inhA* and *pncA*, the mutations in the promoter region along with those in coding regions were associated with resistance to isoniazid and pyrazinamide respectively. In summary, the DRAGdb database is a compilation of all the major MTB drug resistance genes across bacterial species, which allows identification of homoplasy and pleiotropy phenomena of DRAGs.

## Introduction

There is a rise in the use of broad spectrum antibiotics such as rifamycins, aminoglycosides and fluoroquinolones against tuberculosis (TB), as well as common bacterial infections such as gastro-intestinal infections^1–3^. The multi- and extensively drug-resistant (MDR and XDR) *Mycobacterium tuberculosis* (MTB) pose a global threat to public health as new resistance mechanisms are developing and making the treatment for patients prolonged and expensive. Drug resistance is not restricted to TB, but also observed in common bacterial infections such as pneumonia and foodborne infections^4,5^. Genome-wide analysis of MDR and XDR MTB reveals that drug resistance arises due to mutations in the gene and/or the promoter region. Drug resistance associated mutations are linked to increasing drug efflux, modifications of the drugs or their targets^6–8^. The accessibility to next-generation sequencing technologies and characterization of bacteria specific drug resistance allows the extensive study of other pathogenic bacteria as well^9–11^. Antibiotic resistance mutations specific to pathogenic bacteria are available. The Infectious Diseases Society of America has grouped *Enterococcus faecium, Staphylococcus aureus, Klebsiella pneumoniae, Acinetobacter baumannii, Pseudomonas aeruginosa and Enterobacter spp*. as ESKAPE pathogens that are capable of ‘escaping’ the actions of antibiotics thereby developing antibiotic resistance^12^. The ESKAPE pathogens are the leading cause of Hospital-Acquired Infection (HAI) or nosocomial infection^13^. Thus, it is important to understand the drug resistance mutations across ESKAPE species against tuberculosis drugs. Three major databases, Tuberculosis Drug Resistance Mutation database (TBDReaMDB), MUtation BioInformatics Identification (MUBII-TB-DB), Tuberculosis Drug resistance Database (TBDR) are currently available for mutations associated with drug resistance in MTB^14–16^. TBDReaMDB lists information on mutations in 51 genes across both first and second line TB drugs^14^. The major drawback of this database is that it has not been updated after 2009. Other databases such as MUBII-TB-DB and TBDR cover only a small set of genes^15,16^.

Prolonged usage of broad spectrum antibiotics against TB may affect the lung microbiome as well as the intestinal microbiome, which are connected by the “gut-lung axis”^17–20^. In addition, there may be potential horizontal transfer of antibiotic resistant genes in the human microbiome^21–23^. Therefore, there is a need to combine the information from all organism-specific studies into a single platform to have a complete idea of antibiotic resistance associated mutations across bacterial species. In order to facilitate the characterization of mutations in Drug Resistance Associated Genes (DRAGs) across bacterial species, we present DRAGdb, a manually curated database that has enlisted DRAG mutations across bacterial communities focusing on drugs used to treat tuberculosis. DRAGdb provides mutation information related to 6 drugs, a few of which are broad spectrum antibiotics and 12 associated genes across bacterial species including MTB, ESKAPE and other pathogens such as *Escherichia coli* and *Salmonella enterica*. It also provides drug resistance patterns of non-pathogenic bacteria including *Staphylococcus epidermidis* and *Bifidobacterium species*^24,25^. The mutational gene data analysis of DRAGdb highlights the concepts of homoplasy and pleiotropy^26^. Homoplasy is described as a phylogenetic event when a resistance determining mutation arises in phylogeny under selection pressure across species or strains^27^. Another major phylogenetic event occurs when a resistance determining mutation causes pleiotropic effects on resistance to other drugs in a bacteria due to resistance selection^28^. In summary, DRAGdb is a manually curated database of drug resistant genes of bacteria with a focus on TB drugs, which reveals that at least 6 genes carry drug resistance mutations across bacterial species, whereas some drug resistance genes are specific to *Mycobacterium* species.

## Results

### Overview of DRAGdb

#### Database content

DRAGdb is a database of mutational information of DRAGs across MTB clinical strains, ESKAPE bacteria and other pathogenic and non-pathogenic bacterial species with special reference to MTB H37Rv. A systematic curation of mutations found in drug resistant bacteria from existing literature was compiled to create the database. Each mutation entry comprises of organism name, gene name and corresponding identifier from Ensembl Bacteria database, the nucleotide position, the nucleotide change, the amino acid codon position, the codon change, the type of mutation at the amino acid level, the sequencing method used to detect the mutation, the strain of the bacterial species, the geographical location of the sample and PubMed identifier of the literature referred to. The PROVEAN scores predicting the functionality of gene-mutations in different bacteria were added in DRAGdb and the full list of each entry is available in Supplementary File. 2. DRAGdb contains **4653** mutation entries associated with 12 genes and 6 drugs across 126 bacterial species.

### DRAGdb statistics

The basic statistics of DRAGdb is shown in Tables 1 and 2. In Table 1, the MTB gene mutations were compared with existing MTB mutation databases such as TBDReaMDB and MUBII-TB-DB. It was observed that DRAGdb has comparatively higher numbers of mutations for each gene than the other two databases. Table 2 includes MTB genes that were also observed in ESKAPE pathogens and other pathogenic and non-pathogenic bacterial species.

**Table 1:** *Mycobacterium tuberculosis* (MTB) gene mutations reported in DRAGdb compared with TBDReaMDB and MUBII-TB-DB.

Drugs	Genes	No. of mutations			No. of Novel mutations* in DRAGdb
		DRAGdb	TBDReaMDB	MUBII-TB-DB	
Ethambutol	*embA,B,C*	273	11	—	56
Fluoroquinolone	*gyrA*	105	17	17	1
	*gyrB*	69	18	16	—
Isoniazid	*inhA*	30	13	11	17
	*katG*	542	273	—	55
Pyrazinamide	*pncA*	1200	278	277	125
Rifampicin	*rpoB*	710	133	130	32
Streptomycin	*gidB*	178	21	—	37
	*rpsL*	113	16	—	6
	*rrs*	201	25	7	26

*Reported only in DRAGdb.

**Table 2:** ESKAPE and other bacterial species gene mutations reported in DRAGdb.

Drugs	Genes	Bacterial Pathogens	No. of mutations
Fluoroquinolone	*gyrA*	ESKAPE	41
		Others	282
	*gyrB*	ESKAPE	39
		Others	168
Rifampicin	*rpoB*	ESKAPE	73
		Others	346
Streptomycin	*gidB*	ESKAPE	2
		Others	116
	*rpsL*	ESKAPE	4
		Others	129
	*rrs*	ESKAPE	—
		Others	13

### Mutation trends from DRAGdb

**Figure 1:**
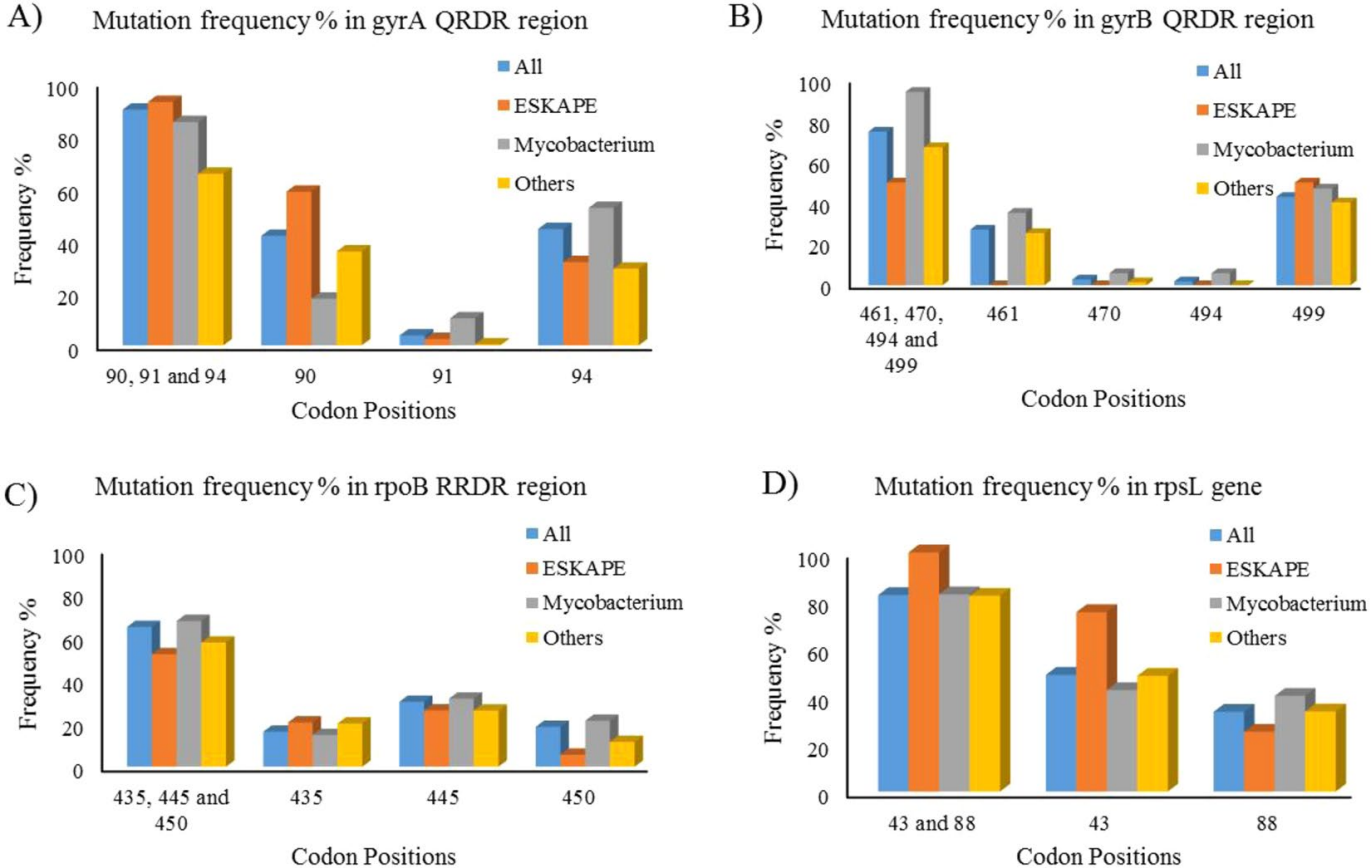
(**A**–**C**) The frequency plots for gyrA, *gyrB* and *rpoB* respectively show mutational frequencies in the top codon positions among all antibiotic resistance determining region codons in each gene. The frequency bars are plotted for ESKAPE pathogens, *Mycobacterium species* and all other bacterial species in each gene. (**D**) The frequency plot of *rpsL* shows mutational frequency of top codon positions among all reported codon positions of mutation. RRDR stands for Rifampicin Resistance Determining Region; QRDR stands for Quinolone Resistance Determining Region.

The literature survey led to compilation of mutational data across different bacterial species for the genes such as *gidB*, *gyrA*, *gyrB*, *rpoB*, *rpsL* and *rrs* (Table 3**)**. Mutations in these genes associated with drug resistance were observed in different bacterial species. However, the genes *inhA*, *katG*, *embA*, *embB*, *embC* and *pncA* are specific to *Mycobacterium species*. The genes associated with drug resistance across different bacterial species, were re-numbered using multiple sequence alignment at codon level with reference to MTB H37Rv, in order to obtain the most frequently mutated codon positions. The frequencies of important drug resistance associated mutations with positions at codon level are represented in bar plots for *gyrA*, *gyrB*, *rpoB*, and *rpsL* in Fig. 1(A–D).

**Table 3:** Genes associated with resistance across a variety of organisms and resistance to a number of drugs.

Gene	No. of organisms	No. of drugs
*gidB*	13	01
*gyrA*	51	14
*gyrB*	41	13
*rpoB*	62	15
*rpsL*	37	04
*rrs*	07	08

### Common set of drug resistance genes across bacterial species

DRAGdb focuses on drugs associated with TB treatment regimens, and lists mutations in associated genes across bacterial genera. It also lists drug resistance genes that are specific to MTB. MTB is relatively “young” from an evolutionary standpoint. It does not carry plasmids and is thus thought not to be engaged in horizontal gene transfer. However, it was observed that mutations in DRAGs of MTB and other bacterial species including ESKAPE pathogens, and other pathogenic and non-pathogenic bacteria, occur usually at the same codon position.

#### gidB

*gidB* also known as *rsmG*, was found to be associated with streptomycin resistance across 13 bacterial species including *Mycobacterium species*, an ESKAPE pathogen i.e. *S. aureus* and other bacteria.

#### gyrA

DRAGdb lists gyrA mutations associated with resistance to second and third generation fluoroquinolones, nalidixic acid and triclosan. *gyrA* mutations were found in 42 bacteria including different *Mycobacterium species*, all 6 ESKAPE pathogens and other bacterial species. The frequency plot of 3 important mutations in the Quinolone Resistance Determining Region (QRDR) of *gyrA* at codon positions 90, 91 and 94 is shown in Fig. 1A and the data is shown in Supplementary File 1: Table S1. The data shows that mutation at the 90^th^ codon position was more dominant in ESKAPE pathogens whereas mutations at the 91^st^ and 94^th^ codon positions occurred more frequently in drug resistant MTB.

#### gyrB

Similar to *gyrA*, *gyrB* was also related to fluoroquinolone resistance. DRAGdb indicates that similar to *gyrA*, most of the *gyrB* mutations were associated with resistance to nalidixic acid and various fluoroquinolones. However, some *gyrB* mutations were associated with resistance to aminocoumarins, a group of gyrase inhibitors which include novobiocin and coumermycin. *gyrB* mutations were found in 36 bacteria including different *Mycobacterium species*, 5 ESKAPE pathogens and other bacterial species. The frequency plot of 4 important mutations in the QRDR of *gyrB* is shown in Fig. 1B and the data is shown in Supplementary File 1: Table S2. The codon at the 499^th^ position was most frequently mutated in ESKAPE, MTB as well as other drug resistant bacteria.

#### rpoB

DRAGdb indicates that mutations in *rpoB* were not only responsible for resistance to the rifamycin class of drugs including rifampicin, rifabutin, rifalazil, rifapentine and rifaximin, but also resistance to 10 other drugs of various drug families in 62 bacterial species including *Mycobacterium species*, the ESKAPE pathogens *Acinetobacter baumannii, Enterococcus faecium, Pseudomonas aeruginosa, Staphylococcus aureus* and many other bacteria. The frequency plot of 3 crucial mutations in the Rifampicin Resistance Determining Region (RRDR) of *rpoB* (as shown in Fig. 1C and in Supplementary File 1: Table S3) shows that mutations at codon positions 435, 445 and 450 exerted an additive effect on drug resistance. Thus no single dominant mutation is alone responsible for resistance to rifamycins in MTB and ESKAPE.

#### rpsL

*rpsL* is primarily associated with streptomycin resistance. However, mutations in r*psL* also cause resistance to other aminoglycosides such as kanamycin, amikacin and paromomycin. *rpsL* mutations were present across 37 bacteria including *Mycobacterium species*, an ESKAPE pathogen *Kleibsella pneumoniae* and other bacterial species. The frequency plot of two dominant drug resistance associated mutations in *rpsL* at codon positions 43 and 88 is shown in Fig. 1D and the data is shown in Supplementary File 1: Table S4.

#### rrs

*rrs* encodes 16S rRNA in bacteria and is associated with streptomycin resistance. DRAGdb shows its involvement in resistance to 5 other aminoglycosides as well. Mutations in *rrs* were found in 7 bacterial species. No mutation has been reported for the ESKAPE pathogens.

### Homoplasy and pleiotropy

**Figure 2:**
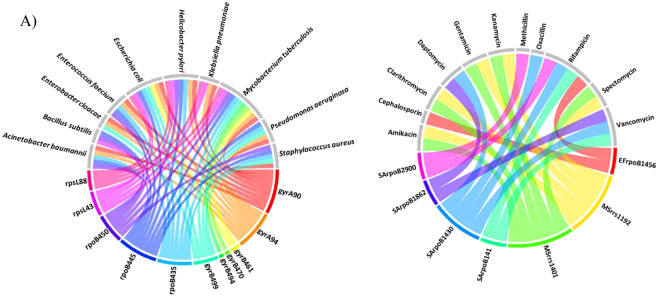
(**A**) The circular plot depicts examples of Homoplasy observed in DRAGdb. Rainbow colored chords connect amino acid positions of the genes (rainbow colored grids) to bacteria (grey colored grids) that have the same point mutation in a specific position associated with resistance to the same drug.The grid name pattern is “gene + codon position”. (**B**) The circular plot depicts examples of Pleiotropy observed in DRAGdb where rainbow colored chords connect nucleotide positions of the genes of specific bacteria (rainbow colored grids) to drug names (grey colored grids) showing that a single nucleotide mutation causes multiple drug resistance. The grid name pattern is “abbreviation of bacterial name + gene + nucleotide position”. SA stands for *Staphylococcus aureus*, MS stands for *Mycobacterium smegmatis* and EF stands for *Enterococcus faecium*. The circular plots were drawn using circlizeR package in R.

Multiple sequence alignments of the protein sequences corresponding to each gene across the reported bacteria were performed as shown in Supplementary File 1: Fig. S1(A–D). Interestingly, in some genes such as *rpoB*, *gyrA*, *gyrB*, *gidB* and *rpsL* similar points of mutations associated with drug resistance, were observed across bacterial species. This could be due to common mechanisms associated with the bacterial response to an antibiotic/drug^29^. Such occurrence of identical genotypes across drug resistant bacterial species is termed here as homoplasy. The MTB H37Rv numbering system was used as reference in our analysis. An example of homoplasy is a point mutation, **Asp to Asn** in *rpoB* at codon position 435 (MTB numbering). This mutation was found in 12 bacterial species including MTB, ESKAPE pathogens such as *Actinobacter baumannii* and *S. aureus*, other pathogenic bacteria including *Helicobacter pylori* and *Haemophilus influenza* and non-pathogenic bacteria, for example, *Deinococcus radiodurans* and *Streptomyces lividans*. In Fig. 2A, the circular plot illustrates some examples of homoplasy events in mutated codon positions across some of the reported bacterial species.

The data curated for DRAGdb also indicates in some bacteria, the presence of cross resistance towards multiple drugs due to a single point mutation. This phenomenon in which a single locus influences resistance to two or more distinct drugs is defined here as pleiotropy. Some of the mutations in *rpoB* across bacterial species were known to be associated with resistance to rifampicin and/or other rifamycins. However, an instance was found in *S. aureus* where mutation in ***rpoB*** at codon position 477 and nucleotide position 1430 (*S. aureus* numbering) was responsible for resistance to rifampicin, daptomycin, vancomycin and oxacillin^30^. In Fig. 2B, the circular plot provides examples of nucleotide positions in genes in specific organisms where a single point mutation is associated with multi-drug resistance.

### Drug resistance genes specific to MTB

**Figure 3:**
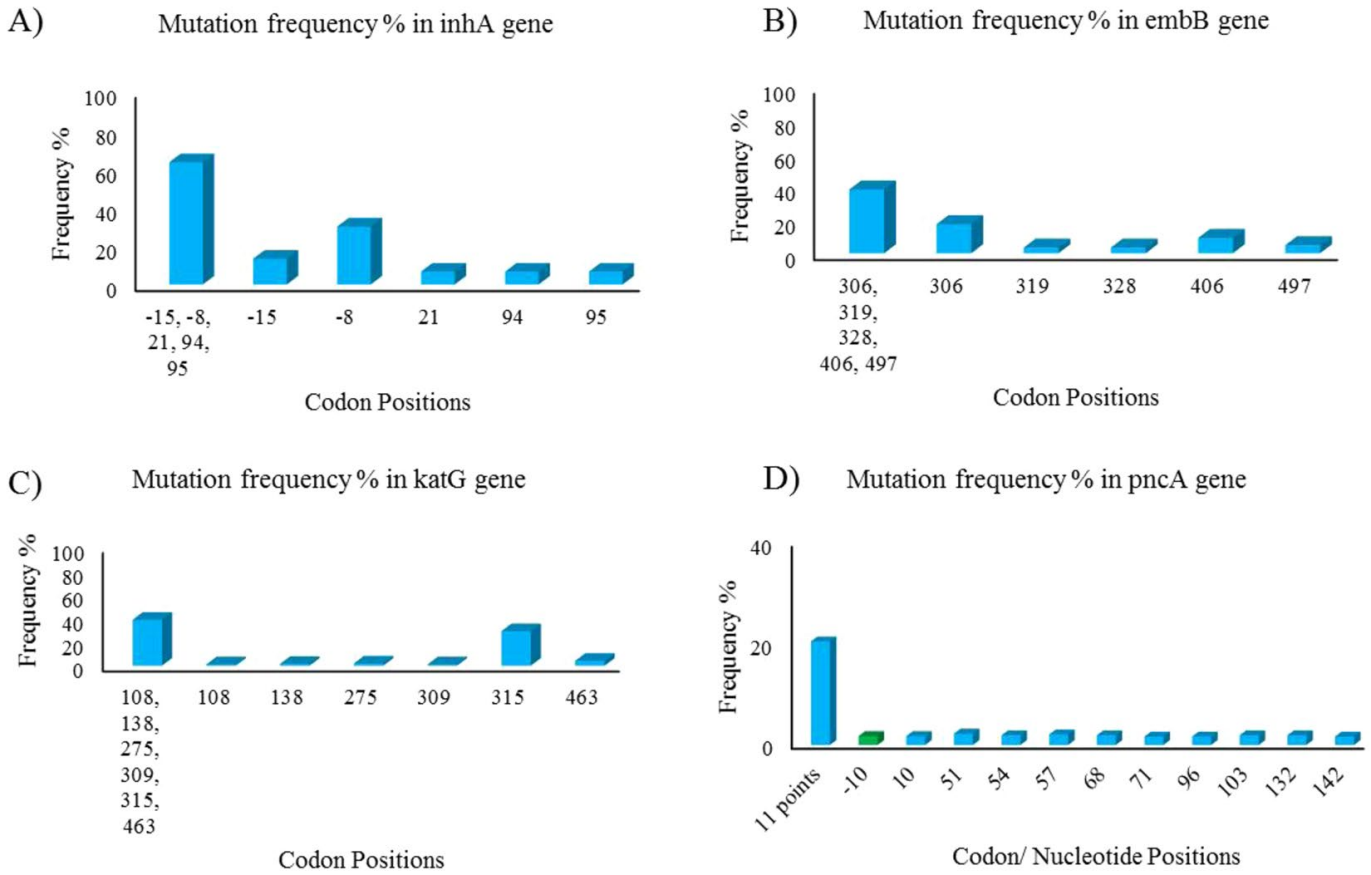
(**A**–**D**) The frequency plot of *inhA*, *embB*, *katG* and *pncA* respectively show mutational frequency of the top codon [**cyan**] or promoter nucleotide [**green**] positions among all reported mutation points in each gene. The frequency bars are plotted for each gene in all *Mycobacterium species*. *The 11points in* (**D**) *includes -10, 10, 51, 54, 57, 68, 71, 96, 103, 132 and 142*.

MTB is assumed to engage very little in horizontal gene transfer and thus considered inert or relatively young in evolutionary terms^31^. It also has an additional layer in its outer membrane composed of novel lipids and polysaccharides such as mycolic acid that makes it an acid fast bacterium^32^. The frequency analysis of 4 *Mycobacterium* specific genes with mutation entries, namely; *inhA*, *embB*, *katG* and *pncA* are shown in Fig. 3(A–D) and numbers are shown in Supplementary File 1: Tables S5–S8. Other than *katG*, all three lacked specific drug resistance determining regions. Drug resistance associated mutations were present all through their coding and non-coding (promoter) regions. Mutation type distribution in MTB specific genes namely *inhA*, *embB*, *katG* and *pncA* is shown in Supplementary File 1: Tables S9-S12 and Supplementary File 1: Figures S2(A–D). It was observed that overall non-synonymous mutations in genic regions dominated over other types of mutations. However, in *inhA* and *pncA*, mutations in promoter regions were also associated with drug resistance.

#### inhA

*inhA* codes for enoyl-ACP reductase and is the primary target of the first-line tuberculosis drug isoniazid^33^. Mutations in the -8 and -15 positions in the promoter region of *inhA* were found in 44% of the isoniazid resistant clinical isolates of *Mycobacterium species*. The frequency plot is shown in Fig. 3A and the data is shown in Supplementary File 1: Table S5. The distribution of mutation types in *inhA* associated with isoniazid resistance is shown in Supplementary File 1: Figure S2A and Table S9.

#### embB

*embB* codes for arabinosyl transferase, an enzyme that plays a role in the polymerization of arabinose into the arabinan of arabinogalactan^34^. It is one of the primary targets of the first line tuberculosis drug ethambutol. Ethambutol inhibits the transfer of arabinogalacton to the cell wall. Mutations in codons 306 and 406 were found in 25% of the ethambutol resistant MTB as shown in Fig. 3B. The data is shown in Supplementary File 1: Table S6. The mutations were mainly observed in the coding region of *embB* as shown in Supplementary File 1: Figure S2B and Table S10.

#### katG

*katG* encodes for a bifunctional enzyme with both catalase and peroxidase activity. It plays a role in protecting *Mycobacterium* against toxic reactive oxygen species as well as in activating the first line drug isoniazid^35,36^. Mutations in 6 codon positions taken together account for 40% of the drug resistant clinical isolates as shown in Fig. 3C. The data is shown in Supplementary File 1: Table S7. Mutation at codon 315 was found in 30% of drug resistant MTB. The distribution of mutation types in *katG* associated with isoniazid resistance is shown in Supplementary File 1: Figure S2C and Table S11.

#### pncA

*pncA* gene codes for pyrazinamidase, which converts the first line tuberculosis drug, pyrazinamide into its active form, pyrazinoic acid^37^. Mutations in *pncA* and its promoter region results in resistance to pyrazinamide. On comparing mutations from pyrazinamide resistant clinical strains of *Mycobacterium species*, it was observed that the mutations were scattered throughout the promoter and the coding region. Overall, 11 sites of mutation accounted for 21% of the mutations in clinical isolates of *Mycobacterium* species. This is shown in Fig. 3D and data is given in Supplementary File 1: Table S8. The mutations in *pncA* were observed to be diverse in nature (shown in Supplementary File 1: Figure S2D and Table S12).

### Comparison with other databases and tools

**Figure 4:**
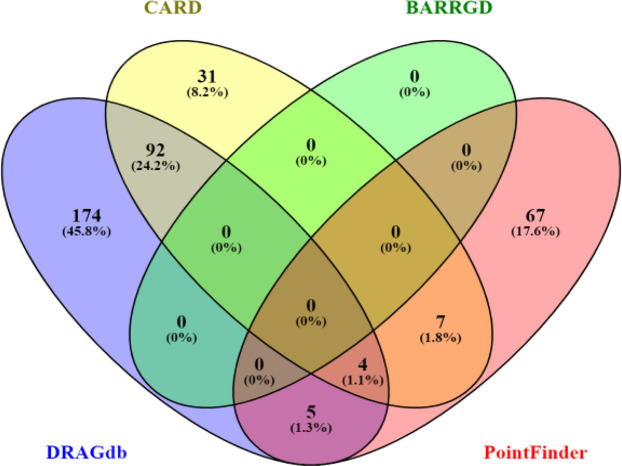
The venn diagram for the comparison of rpoB mutations in MTB and ESKAPE pathogens along with *Mycobacterium leprae, Escherichia coli, Enterococcus faecalis* among CARD, BARRGD, PointFinder and DRAGdb. BARRGD has rpoB mutations of Proteobacteria at phylum level thus it has no common entry at species level.

There are several antibiotic resistance related databases as listed in Supplementary File 1: Table S13 obtained from PubMed literature search. The contents of the databases such as the bacterial species focused, data types, availability of mutation data were thoroughly studied and compared. Out of these 17 databases, three were beta-lactamases related resources^38–40^, five were specific to single species such as uCARE^41^ is for *E.coli* and TBDB, ReSeqTB^42^, TBDReaMDB and MUBII-TB-DB were dedicated to MTB. A comparison of TBDreamDB and MUBII-TB-DB, two well-known databases for mutations associated with drug resistance in MTB with DRAGdb is presented in Table 1. DRAGdb lists a higher number of gene mutations. There were nine multispecies antimicrobial databases. Among them, MEGARes^43^, BacMet^44^, Resfams^45^ and Pathosystems Resource Integration Center (PATRIC)^46^ contain bacterial genome and drug resistance genes but no mutation data is available in them. There were two deprecated databases such as ARG-ANNOT^47^ and ARDB - Antibiotic Resistance Genes Database^48^, however, ARG-ANNOT gene list is incorporated in MEGARes and ARDB is upgraded as CARD^49^. Finally it was observed that only three databases contain updated drug resistance causing mutation data across species namely; CARD, BARRGD^50^ and PointFinder^51^. The mutation data available in these three databases were downloaded for further analysis. A case study to compare the rpoB gene mutations associated with drug resistance in MTB and ESKAPE pathogens along with *Mycobacterium leprae, Escherichia coli, Enterococcus faecalis* present in these three databases and DRAGdb was done. A venn diagram in Fig. 4 shows that DRAGdb had 174 unique SNPs in rpoB gene compared to other three databases. The unique list of rpoB mutations in DRAGdb as shown in supplementary File. 3 comprises mainly of mutations in bacteria like *Enterococcus faecium, Acinetobacter baumannii, Pseudomonas aeruginosa* and *Enterococcus faecalis* that were only available in DRAGdb and few mutation points in other bacteria also. There are also some tools for the prediction of antibiotic resistance genes such as meta-MARC that predicts drug resistance from metagenomics data^52^, and AMRFinder that uses hidden Markov model of BARRGD sequence database to identify the genes related to drug resistance^53^. DRAGdb use its own drug resistance associated gene sequence database at bacterial species level for Basic Local Alignment Search Tool (BLAST) search. Thus it allows users to identify the best hit mutant sequence at species level. This cannot be achieved with AMRFinder.

### Utility and limitations of DRAGdb

**Figure 5:**
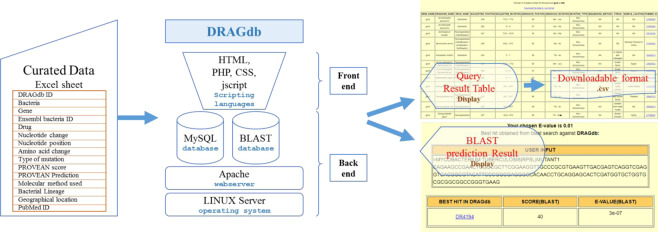
Schematic architecture of DRAGdb online database.

The benefit of DRAGdb is that it provides information on antibiotic resistance related mutations across various bacterial species in a single platform. As shown in the schematic diagram of DRAGdb in Fig. 5, in addition to browsing the mutation database, the BLAST tool is integrated for prediction of drug resistance from a query sequence. Compared to existing databases, DRAGdb contains higher numbers of, as well as unique drug resistance associated gene mutations as shown in Table 1. The caveat of this version of DRAGdb, is that all the double or multiple mutations in a drug resistant gene are considered as separate entries for each species and thus the overall effect of all drug resistant mutations is not presented in a comprehensive manner in a specific search. Further, the effect of mutations in multiple genes in MDR, for example *gyrA* and *rpoB*, cannot be obtained in a single search. However, the BROWSE page of DRAGdb allows users to get all the information in tabular format.

## Discussion

In the recent past, due to the increasing availability of next generation technologies, a large number of studies have been carried out to unravel the specificity of drug resistance in many pathogenic bacteria. Here, we describe DRAGdb a database that contains mutational data across MTB, ESKAPE pathogens, other pathogenic bacteria such as those causing sexually transmitted infections (*Neisseria gonorrhoeae*), foodborne infections (*Campylobacter jejuni*), skin infections (*Streptococcus pyogenes*), and non-pathogenic organisms such as *Bifidobacterium species*. Compared to the existing TB mutation databases such as TBDReaMDB, MUBII-TB-DB and TBDR, DRAGdb data carries more extensive mutational data^14–16^. DRAGdb data indicates the presence of similar mutation patterns in 6 drug resistant genes, namely *rpoB*, *gyrA*, *gyrB*, *gidB*, *rrs* and *rpsL* across bacterial ecosystems, that in turn highlights the drawbacks of using broad spectrum antibiotics for prolonged treatment of diseases such as tuberculosis^1–5^. We suggest that prolonged exposure to drugs required for the treatment of TB, leads to occurrence of resistance across bacterial populations in the gut microbiome that may hinder treatment of other bacterial infections^20^. However, on a positive note, identifying a common cause of resistance across a wide range of bacterial species opens up the possibility of designing diagnostic tools and identifying specific drug targets for a wide range of bacterial infections. The data presented here points to the occurrence of resistance in many pathogenic bacterial species along with the MTB clinical strains, the ESKAPE pathogens and commensal or non-pathogenic bacteria. There is an urgent need to focus on the purportedly under-rated pathogens which may cause severe health problems in the near future due to homoplasy and pleiotropy. DRAGdb also indicates that for the MTB specific drug resistance genes *pncA*, *inhA*, *katG* and *embA, B, C* in addition to the non-synonymous mutations in coding region, the non-coding regions also play important roles associated with drug resistance. This brings an additional layer of complexity to the mechanisms of drug resistance. Further, a systematic analysis of mutations responsible for drug resistance in a bacterial community against specific drugs, is required to understand the evolution in drug resistance genes in response to drug exposure.

## Conclusions

Antibiotic/drug resistance is a natural phenomenon in microbial populations and is a global health threat making the usage of antibiotics to treat life threatening infections such as tuberculosis and pneumonia less and less effective. Tuberculosis treatment requires broad spectrum antibiotic classes such as rifamycins, aminoglycosides and fluoroquinolones that are also extensively used against other bacterial infections. To contribute towards the analysis of the development of antibiotic/drug resistance we have developed the DRAGdb database. It is a free online repository of mutations in genes associated with broad spectrum antibiotics across *Mycobacterium species*, ESKAPE pathogens and other pathogenic and non-pathogenic bacteria, along with MTB specific drug resistance genes associated with drugs such as pyrazinamide, isoniazid and ethambutol. The database can be easily searched and browsed at http://bicresources.jcbose.ac.in/ssaha4/drag. DRAGdb also includes a BLAST search option to predict drug resistance related mutations. Comparison and analysis of mutations in DRAGs across bacterial species give a clear indication of two phylogenetic phenomena namely homoplasy and pleiotropy. Six genes (*gidB*, *gyrA*, *gyrB*, *rpoB*, *rpsL* and *rrs*) were associated with drug resistance not only in MTB but also in ESKAPE and other bacterial pathogens. For these genes, we analyzed coding regions using MSA where MTB H37Rv was used as reference genome. Some genes (*inhA*, *embB*, *katG* and *pncA*) were specific to MTB. The promoter regions of *inhA* and *pncA* were involved in drug resistance along with their genic regions. The study clearly indicates that under the stress of drug exposure, the response is not random. Instead it follows a defined pattern across bacterial communities.

## Methods

### Database implementation

DRAGdb comprises of a single table where each mutation entry is uniquely identified with DRAGDB_ID as the primary key. The NUCLEOTIDE_POSITION, NUCLEOTIDE_CHANGE, AMINOACID_POSITION, AMINOACID_CHANGE define the mutation point at both levels. The PUBMED_ID provides PubMed identifier, hyperlink to PubMed database and ENSEMBL_BACTERIA_ID provides the gene identifier.

DRAGdb was developed using the Apache HTTP 2.2.15 web server and MySQL 5.1.69. The PHP 5.3.3, HTML, JavaScript and CSS were used to build the web interfaces of the database. The PHP-based web interfaces execute the SQL queries dynamically. It is freely accessible at http://bicresources.jcbose.ac.in/ssaha4/drag.

### Data curation

The PubMed database (till March 2018) was searched for studies that reported at least one mutation in *rpoB*, *pncA*, *inhA*, *katG*, *embA*, *embB*, *embC*, *gidB*, *rpsL*, *rrs*, *gyrA* and *gyrB* associated with resistance to rifampicin, pyrazinamide, isoniazid, ethambutol, streptomycin and fluoroquinolones respectively in MTB, ESKAPE and other bacterial species. The literature was searched using advance search option of PubMed with the terms: “Gene name (Abstract/title) AND Resistance (Abstract/title) AND mutation (Abstract/title) AND/ NOT tuberculosis (Abstract/title)”. The combination of search terms helped to obtain instances with cross resistance and multiple resistances. In total, 2548 unique publications were obtained from this search. The publications that were missing full English text in public domain, or lacked relevant data or had ambiguous data were filtered out. Around 604 publications were systematically reviewed to obtain mutational information. All the mutations described in drug resistant bacterial strains in the literature were manually read, further curated and compiled in the database. The devised methodology is given as workflow in Supplementary File 1: Figure S3.

### Mutation data analysis with reference to MTB H37Rv

All the gene mutations reported in the literature across bacterial species have different numbering systems (NS) thus leading to genetic location inconsistency and conflict. One of the examples of NS discrepancy is of *gyrA* in MTB, for which 4 different NS were found in the literature^54^. For better understanding and comparison across species of a single gene, *Mycobacterium tuberculosis* H37Rv was selected as reference organism, further multiple sequence alignment (MSA) was performed at amino acid codon level for each drug resistance gene to have single numbering system across all organisms. MSA was performed on on-line Clustal Omega platform using default iterated mBed-like Clustering Guide-tree^55,56^. The rational for choosing MTB as reference genome was due to the fact that exposure of 3–6 antibiotics including broad spectrum antibiotics during TB treatment for 6 months results in known multiple drug resistance phenotypes. The MSA of the regions of interest for genes such as *gyrA*, *gyrB*, *rpoB*, and *rpsL* were shown in Supplementary File 1: Figures S1(A–D). The common reference number at the amino acid codons level of drug resistance genes across bacterial species helped in calculating frequency of mutated codon positions in DRAGdb. The frequency percentage was calculated using the following formulae –

$${F}_{xi}=\,\frac{{N}_{xi}}{{\sum }_{i=j}^{t}{N}_{xi}}\times 100$$

where ${F}_{xi}$ is the frequency percentage of ${i}^{th}$ codon or nucleotide position in a gene of ${x}^{th}$ group, $x$ can be all organisms, Mycobacterium, ESKAPE pathogens or other bacteria. ${N}_{xi}$ is the number of mutation entries of ${i}^{th}$ codon or nucleotide position in the gene of ${x}^{th}$ group in DRAGdb. $\mathop{\sum }\limits_{i=j}^{t}{N}_{xi}$ is the total number of mutation entries in the drug resistance determining region (DRDR) of the gene, $j$ is the starting codon or nucleotide position and $t$ is the end codon or nucleotide position of DRDR. The number of mutation entries was calculated based on report of a single mutation across various PubMed literature. We assume that the higher the number of publications reporting a particular drug resistance determining gene mutation, the higher is the confidence of that mutation entry.

### Functional effects of the mutations

The functional effects of the unique SNPs in drug-resistance genes in different bacteria were predicted using PROVEAN webserver with Score thresholds for prediction as of −2.5. The variants with score equal to or below of −2.5 were considered “deleterious”, and the variants with score of above −2.5 were considered “neutral”^57^.

### Blast search

A customized BLAST database was created with wild type and mutated small nucleotide stretches of drug resistance determining regions of associated genes. The mutated sequences were modified wild type sequences with incorporation of single mutations enlisted in DRAGdb. *blastall*, a package for BLAST search was used^58^. *formatdb* utility from that package was used for converting nucleotide FASTA sequences to BLAST database. *blastn* program was used to find similar sequences to query sequences in the BLAST database.

### DRAGdb user interface

The ‘HOME’ page of DRAGdb web interface provides two different search options: 1) keyword search: a single keyword can be searched specific to bacteria, resistant drugs, genes, geographical location or ‘ALL’ option to search in any category. 2) Advance search: three fields are present where bacteria and gene name are mandatory and drug name is optional. Both the search options will generate a table giving details of the mutations related to the search and also provide the number of specific entries. The DRAGdb result pages also contain hyperlinked Ensembl Bacteria IDs, PROVEAN score and PubMed IDs. To keep with the open access policy, the result table can be downloaded by the users. The ‘BROWSE’ page allows users to browse DRAGdb data in three categories: 6 drugs, 12 genes, and 126 bacterial species. It shows the comparison of DRAGdb data with other tuberculosis databases namely, TBDReaMDB and MUBII-TB-DB. The ‘Organisms’ section is further divided into 3 parts: ‘*Mycobacterium tuberculosis’*, ‘ESKAPE’ and ‘others’ which includes other bacterial species. The entries within the three categories are linked to DRAGdb table and provide specific results with details of the gene mutations. The nucleotide BLAST search with customized BLAST database is incorporated in the ‘TOOL’ page to determine whether the users input bacterial gene sequence is drug resistant. Users can define the ‘E-value’ for BLAST operation. The output page shows the user input sequence, the DRAGDB_ID of the best hit, the BLAST score and E-value of the hit. ‘OTHER LINKS’ page is also included to help users find popular TB and antibiotic resistance related databases and webservers. To guide users through DRAGdb, a ‘HELP’ page is also presented in the online web server.

### Data visualization

The bar plots for representation of frequency % of various codon level mutations of drug resistance genes across bacterial species were drawn using Microsoft office excel. The circular plots for representations of homoplasy and pleiotropy were drawn using ‘circlize’ R package^59^.

## Supplementary information

Supplementary file1.

Supplementary file2.

Supplementary file3.

## Data Availability

Data are available at http://bicresources.jcbose.ac.in/ssaha4/drag/browse.php. Supporting figures and tables are included in Supplementary Files 1–3.
