# Supplementary material for: Survey of drug resistance associated gene mutations in Mycobacterium tuberculosis, ESKAPE and other bacterial species
Source: Sci Rep. 2020 Jun 2;10:8957. doi: 10.1038/s41598-020-65766-8 (PMC7265455; doi:10.1038/s41598-020-65766-8)
Supplement: Supplementary file 1 — Supplementary file1. [file 41598_2020_65766_MOESM1_ESM.docx]

**Survey of drug resistance associated gene mutations in *Mycobacterium tuberculosis*, ESKAPE and other bacterial species**

Abhirupa Ghosh^1^^, Saran N^1^^ and Sudipto Saha^1^*

^1^ Division of Bioinformatics, Bose Institute, India.

^ Equal Contribution; *Corresponding Author.

**Abhirupa Ghosh (Email**: abhirupa@jcbose.ac.in)

**Saran N (Email:** nsaran452@gmail.com**)**

***Corresponding Author:**

**Dr. Sudipto Saha**

**Division of Bioinformatics**

**Centenary Campus, Bose Institute**

**P-1/12 C. I. T. Scheme VII-M, Kolkata – 700054, India**

**Email:** [**ssaha4@jcbose.ac.in**](mailto:ssaha4@jcbose.ac.in), [**ssaha4@gmail.com**](mailto:ssaha4@gmail.com)

**Phone: +91-33-25693333**

**Survey of drug resistance associated gene mutations in *Mycobacterium tuberculosis*, ESKAPE and other bacterial species**

Abhirupa Ghosh^1^^, Saran N^1^^ and Sudipto Saha^1^*

^1^Bioinformatics Centre, Bose Institute, Kolkata – 54.

^ Equal Contribution; *corresponding author: [ssaha4@gmail.com](mailto:ssaha4@gmail.com), [ssaha4@jcbose.ac.in](mailto:ssaha4@jcbose.ac.in)

**Supplementary Information**

**Table S1:** The frequency table of **gyrA**.

|  | All Organisms | ESKAPE | Mycobacterium | Others |
| --- | --- | --- | --- | --- |
| 3 points | 89.79592 | 92.68293 | 85.04673 | 65.32846715 |
| 90 | 41.69096 | 58.53659 | 17.75701 | 35.76642336 |
| 91 | 3.790087 | 2.439024 | 10.28037 | 0.364963504 |
| 94 | 44.31487 | 31.70732 | 52.33645 | 29.19708029 |

**Table S2:** The frequency table of **gyrB**.

|  | All Organisms | ESKAPE | Mycobacterium | Others |
| --- | --- | --- | --- | --- |
| 4 points | 74.76636 | 50 | 94.11765 | 67.16418 |
| 461 | 27.1028 | 0 | 35.29412 | 25.37313 |
| 470 | 2.803738 | 0 | 5.882353 | 1.492537 |
| 494 | 1.869159 | 0 | 5.882353 | 0 |
| 499 | 42.99065 | 50 | 47.05882 | 40.29851 |

**Table S3:** The frequency table of **rpoB**.

|  | All organisms | ESKAPE | Mycobacterium | Others |
| --- | --- | --- | --- | --- |
| 3 points | 64.43243 | 51.85185 | 67.16418 | 57.21393035 |
| 435 | 16.10811 | 20.37037 | 14.62687 | 19.90049751 |
| 445 | 29.94595 | 25.92593 | 31.34328 | 25.87064677 |
| 450 | 18.37838 | 5.555556 | 21.19403 | 11.44278607 |

**Table S4:** The frequency table of **rpsL**.

|  | All Organisms | ESKAPE | Mycobacterium | Others |
| --- | --- | --- | --- | --- |
| 2 points | 82.11382 | 100 | 82.4 | 81.81818182 |
| 43 | 48.78049 | 75 | 42.4 | 48.34710744 |
| 88 | 33.33333 | 25 | 40 | 33.47107438 |

**Table S5:** The frequency table of **inhA** in *Mycobacterium species*.

| Codon Position | Mutation Frequency |
| --- | --- |
| 5 points | 63.33333 |
| -15 | 13.33333 |
| -8 | 30 |
| 21 | 6.666667 |
| 94 | 6.666667 |
| 95 | 6.666667 |

**Table S6:** The frequency table of **embB** in *Mycobacterium species*.

| Codon Position | Mutation Frequency |
| --- | --- |
| 5 points | 38.34951456 |
| 306 | 17.47572816 |
| 319 | 3.398058252 |
| 328 | 3.398058252 |
| 406 | 9.223300971 |
| 497 | 4.854368932 |

**Table S7:** The frequency table of **katG** in *Mycobacterium species*.

| Codon position | Mutation Frequency |
| --- | --- |
| 6 points | 38.7037 |
| 108 | 1.111111 |
| 138 | 1.481481 |
| 275 | 1.851852 |
| 309 | 1.111111 |
| 315 | 29.07407 |
| 463 | 4.074074 |

**Table S8:** The frequency table of **pncA** in *Mycobacterium species*.

| Codon position | Mutation Frequency |
| --- | --- |
| 11 points | 20.5 |
| -10 | 1.75 |
| 10 | 1.75 |
| 51 | 2.25 |
| 54 | 1.916667 |
| 57 | 2.083333 |
| 68 | 1.916667 |
| 71 | 1.666667 |
| 96 | 1.666667 |
| 103 | 1.916667 |
| 132 | 1.916667 |
| 142 | 1.666667 |

**Table S9:** The mutation distribution table of **inhA** in *Mycobacterium species*.

| inhA | inhA mutation |
| --- | --- |
| Promoter | 17 |
| Non - Synonymous | 13 |

**Table S10:** The mutation distribution table of **embB** in *Mycobacterium species*.

| embB | embB mutation |
| --- | --- |
| Non - Synonymous | 205 |
| Frameshift | 1 |

**Table S11:** The mutation distribution table of **katG** in *Mycobacterium species*.

| katG | katG mutation |
| --- | --- |
| Non - Synonymous | 482 |
| Frameshift | 33 |
| Synonymous | 14 |
| Deletion | 9 |
| Insertion | 2 |

**Table S12:** The mutation distribution table of **pncA** in *Mycobacterium species*.

| pncA | pncA mutation |
| --- | --- |
| Non-synonymous | 840 |
| Frameshift | 283 |
| Promoter | 45 |
| Deletion | 11 |
| Synonymous | 10 |
| Insertion | 8 |
| Others | 3 |

**Table S13:** The list of antimicrobial databases obtained from PubMed search.

| Serial no. | Database/ Resource | Description | Comment | Gene mutations | No. of total Unique mutations | No. of bacterial species | links |
| --- | --- | --- | --- | --- | --- | --- | --- |
| 1 | Comprehensive Antibiotic Resistance Database (CARD)[^1^](#_ENREF_1) | Curated resource of reference DNA and protein sequences, detection models and bioinformatics tools on the molecular basis of bacterial antimicrobial resistance. | Multiple species  March 2020 release | present | 1468 | 57 | [https://card.mcmaster.ca](https://card.mcmaster.ca/) |
| 2 | Bacterial Antimicrobial Resistance Reference Gene Database[^2^](#_ENREF_2) | Annotated sequence records for representative DNA sequences that encode proteins conferring or contributing to resistance to various antibiotics. | Multiple species  **Database version:** 2020-01-22.1 | Present as allele names | 472 | 7 (other entries are multi-species) | <https://www.ncbi.nlm.nih.gov/pathogens/isolates#/refgene/> |
| 3 | Beta-Lactamase Data Resources  (Lahey beta-lactamase archive)[^3^](#_ENREF_3) | Genotypic and functional characterization of beta-lactamases | deprecated | Not present |  |  | NCBI BioProject PRJNA305729 |
| 4 | PointFinder^[4](#_ENREF_4)^ | Database of ResFinder for WGS-based detection of antimicrobial resistance associated with chromosomal point mutations in bacterial pathogens | Multiple species  PointFinder database: (2019-07-02) | present | 1549 | 7 | <https://cge.cbs.dtu.dk/services/ResFinder/> |
| 5 | ARG-ANNOT[^5^](#_ENREF_5) | recursive database of all known AR genes | the website was no longer supported | NA |  |  | NA |
| 6. | MEGARes  ^6^ | non-redundant compilation of sequences contained in Resfinder (November 2015), ARG-ANNOT (November 2015), the Comprehensive Antibiotic Resistance Database (CARD, v1.0.7), and the National Center for Biotechnology Information (NCBI) Lahey Clinic beta-lactamase archive (December 2015) | Multiple species | Not present |  |  | <https://megares.meglab.org/> |
| 7 | BacMet^[7](#_ENREF_7)^ | resource of antibacterial biocide- and metal-resistance genes | Multiple species | Not present |  |  | <http://bacmet.biomedicine.gu.se/> |
| 8 | ARDB - Antibiotic Resistance Genes Database[^8^](#_ENREF_8) | manually curated database on antibiotic resistance | ARDB is no longer being maintained. All data in ARDB are also found in CARD | Present |  |  | <https://ardb.cbcb.umd.edu/> |
| 9 | Resfams^[9](#_ENREF_9)^ | Resfams is a curated database of protein families and associated profile hidden Markov models (HMMs), confirmed for antibiotic resistance function and organized by ontology. | Resfams profile HMMs were first trained using 2097 unique AR protein sequences from the CARD, the Lactamase Engineering Database (LacED) and Lahey beta-lactamase archive | Not present |  |  | <http://www.dantaslab.org/resfams> |
| 10 | uCARE, a Comprehensive Antibiotic resistance Repository of Escherichia coli[^10^](#_ENREF_10) | integrates gene and genomic data in context to drug resistance in E. coli | E. coli | partial |  |  | <http://www.e-bioinformatics.net/ucare/> |
| 11 | Beta-Lactamase DataBase (BLDB)[^11^](#_ENREF_11) | resource providing structural and functional information focused on beta-lactamase enzymes with a great impact on antibiotic resistance | Species information not available | present | 148 |  | <http://www.bldb.eu/> |
| 12 | CBMAR: a comprehensive β-lactamase molecular annotation resource[^12^](#_ENREF_12) | information useful for molecular and biochemical characterization of each family of β-lactamase | Species information not available | present |  |  | <http://14.139.227.92/mkumar/lactamasedb> |
| 13 | Pathosystems Resource Integration Center (PATRIC)[^13^](#_ENREF_13) | PATRIC provides scientists with (i) a comprehensive bacterial genomics database, (ii) a plethora of associated data relevant to genomic analysis, and (iii) an extensive suite of computational tools and platforms for bioinformatics analysis. | Multiple species | Not present |  |  | <https://patricbrc.org/> |
| 14 | TBDB[^14^](#_ENREF_14) | Standard database for the TBProfiler tool | MTB | present | 1536 |  | <https://github.com/jodyphelan/tbdb> |
| 15 | ReSeqTB^[15](#_ENREF_15)^ | ReSeqTB is curated knowledgebase, of Mycobacterium tuberculosis complex (MTBC) variant data from whole genome sequencing (WGS) with phenotypic drug susceptibility testing (DST) and clinical data. | MTB | present | 246 |  | - <https://platform.reseqtb.org/> |
| 16 | TBDReaMDB^[16](#_ENREF_16)^ | database devoted to drug resistance mutations in TB | MTB | present | 1337 |  | - <https://www.tbdreamdb.com/> |
| 17 | MUBII-TB-DB[^17^](#_ENREF_17) | database that contains a set of Mycobacterium tuberculosis mutations (DNA and proteins) occurring at seven loci: rpoB, pncA, katG; mabA(fabG1)-inhA, gyrA, gyrB, and rrs. | MTB | Present | 618 |  | <https://umr5558-proka.univ-lyon1.fr/mubii/mubii-select.cgi> |


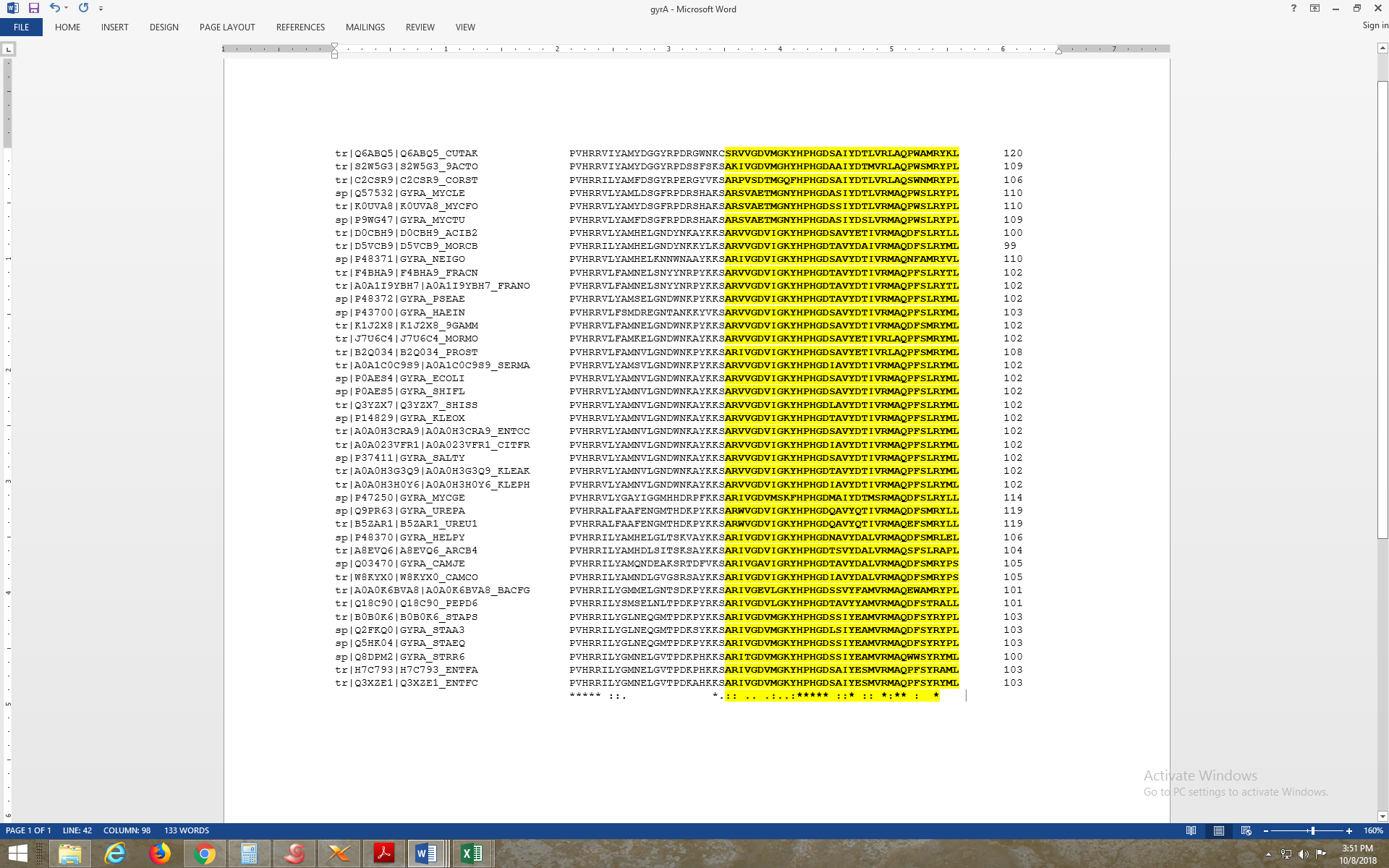


**FigureS1A:** Multiple sequence alignment of **gyrA** across bacterial species. The bold and yellow marked region is **QRDR**.


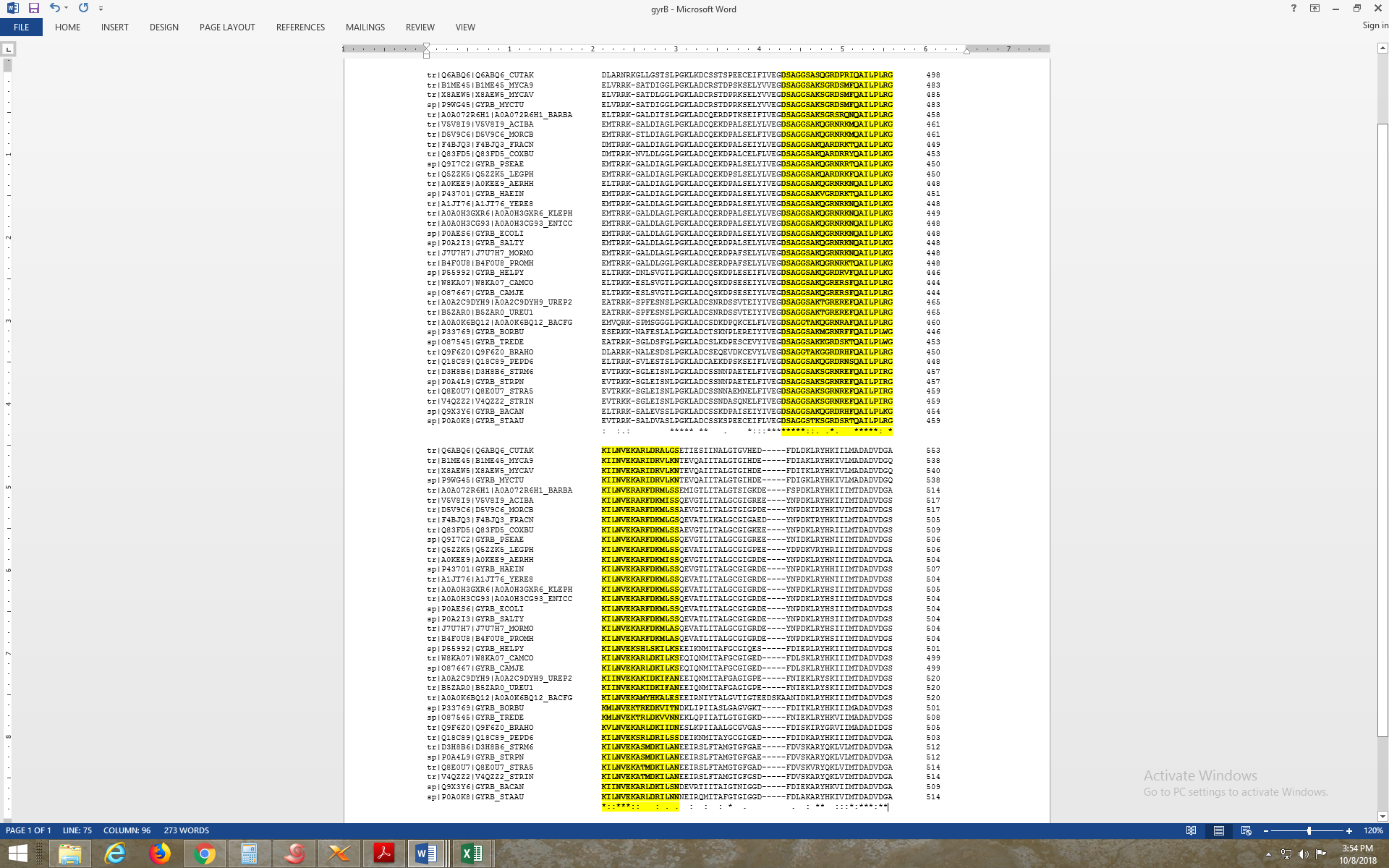


**FigureS1B:** Multiple sequence alignment of **gyrB** across bacterial species. The bold and yellow marked region is **QRDR**.


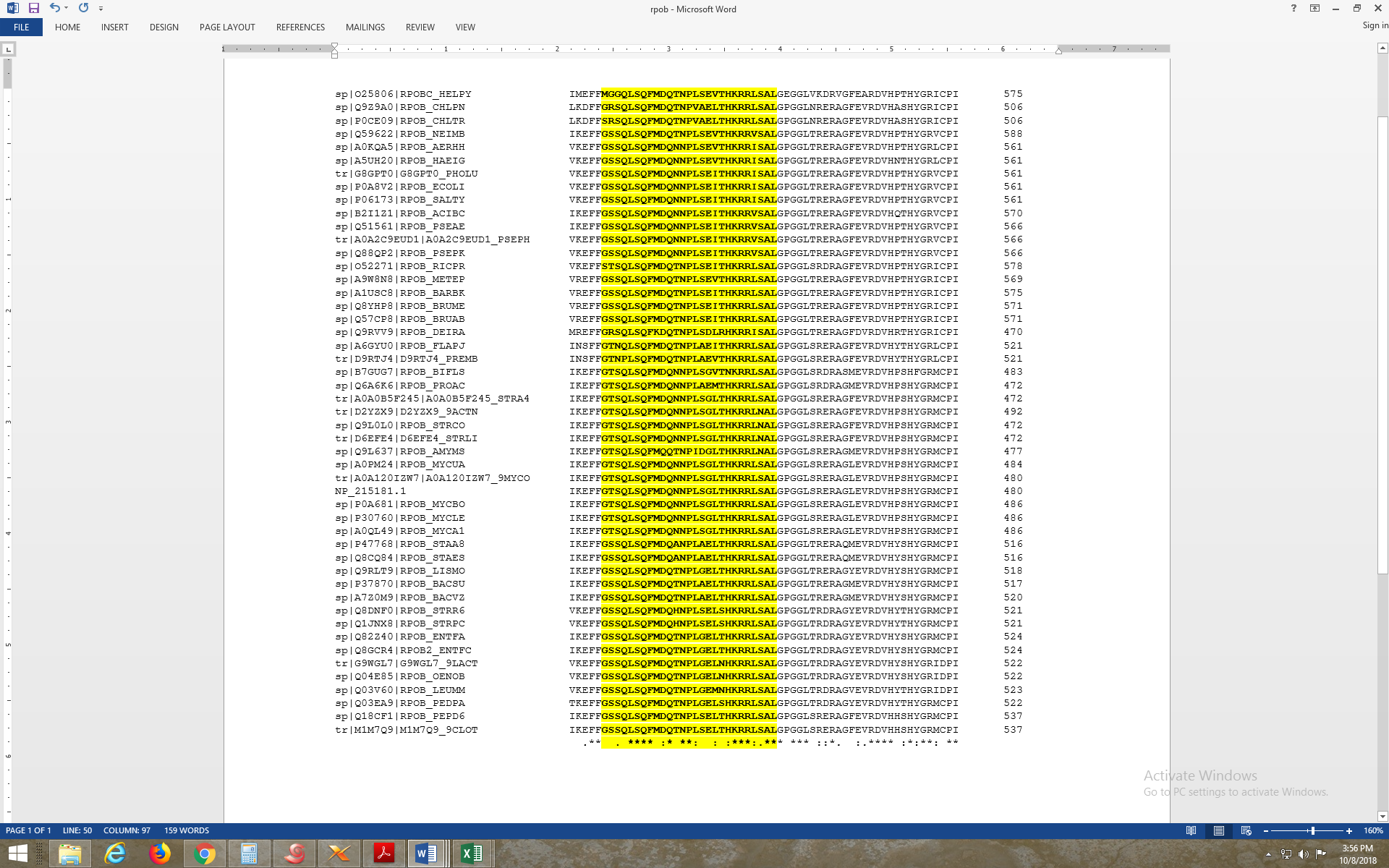


**FigureS1C:** Multiple sequence alignment of **rpoB** across bacterial species. The bold and yellow marked region is **RRDR**.


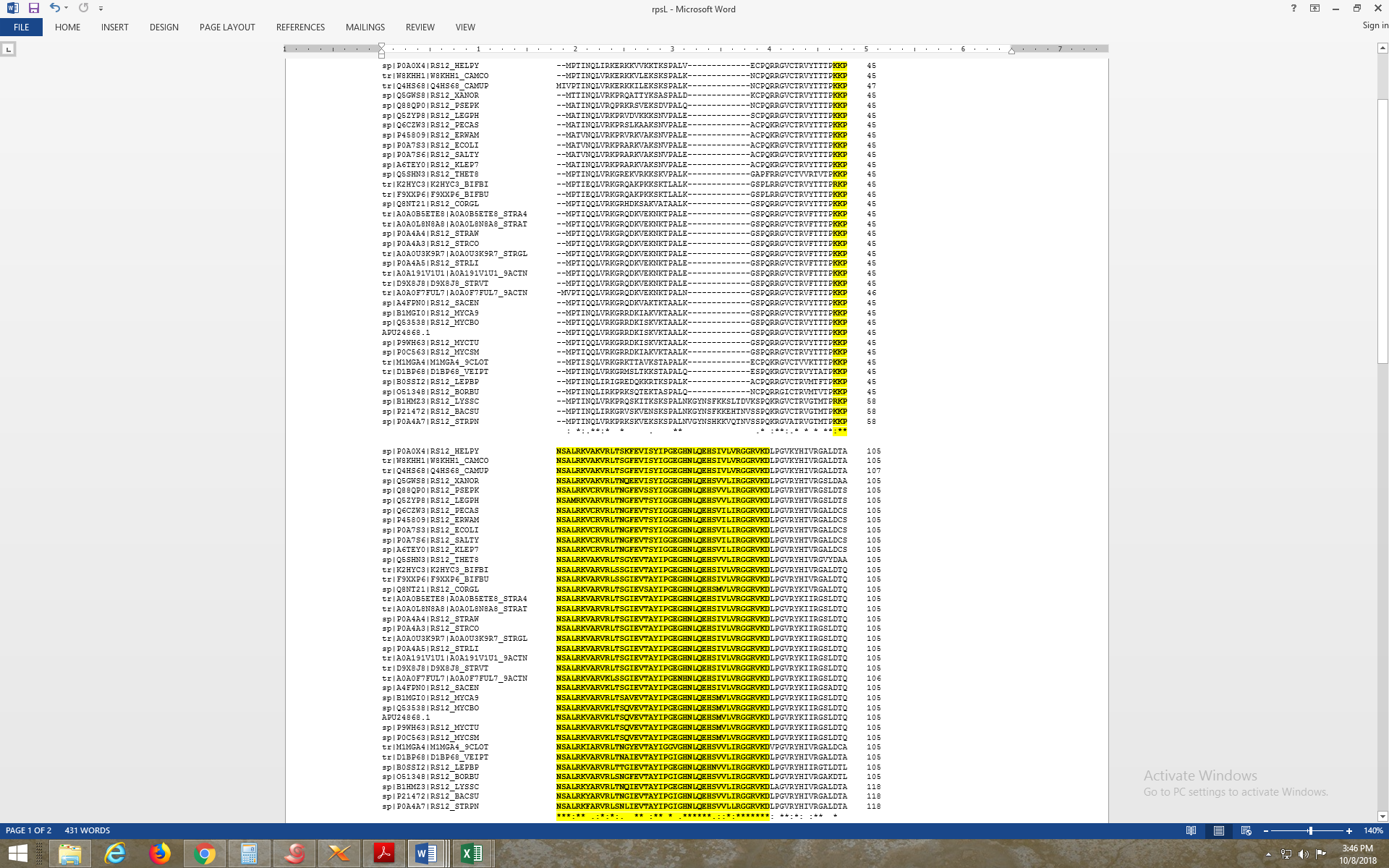


**FigureS1D:** Multiple sequence alignment of **rpsL** across bacterial species. The bold and yellow marked region comprises the 43^rd^ and 88^th^ positions.

**FigureS2A:** The mutation distribution of **inhA** in *Mycobacterium species*.

**FigureS2B:** The mutation distribution of **embB** in *Mycobacterium species*.

**FigureS2C:** The mutation distribution of **katG** in *Mycobacterium species*.

**FigureS2D:** The mutation distribution of **pncA** in *Mycobacterium species*.


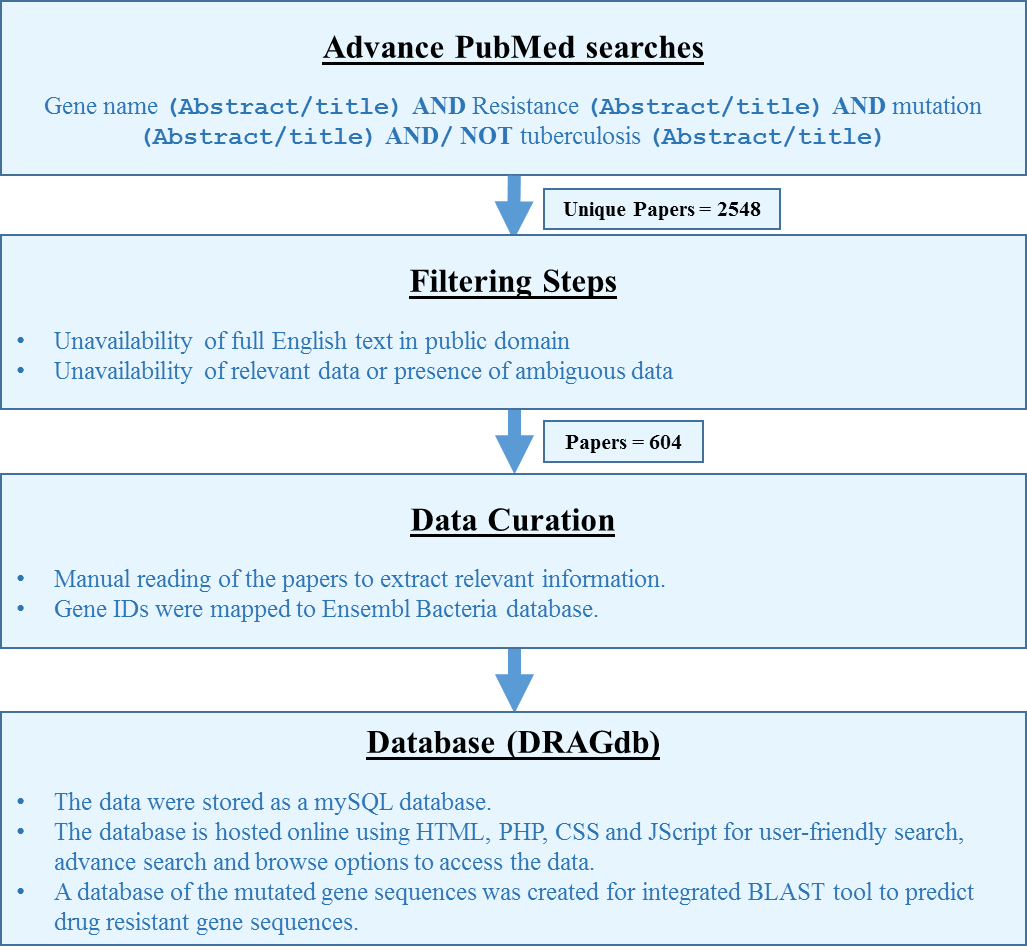


**Figure S3:** The workflow of the devised methodology to develop DRAGdb, analysis of the data and integration of BLAST search. *MSA:multiple sequence alignment.*

**References:**

1 Alcock, B. P. *et al.* CARD 2020: antibiotic resistome surveillance with the comprehensive antibiotic resistance database. *Nucleic Acids Res* **48**, D517-D525, doi:10.1093/nar/gkz935 (2020).

2 [*https://www.ncbi.nlm.nih.gov/pathogens/isolates#/refgene/*](https://www.ncbi.nlm.nih.gov/pathogens/isolates#/refgene/).

3 Bush, K. & Jacoby, G. A. Updated functional classification of beta-lactamases. *Antimicrob Agents Chemother* **54**, 969-976, doi:10.1128/AAC.01009-09 (2010).

4 Zankari, E. *et al.* PointFinder: a novel web tool for WGS-based detection of antimicrobial resistance associated with chromosomal point mutations in bacterial pathogens. *J Antimicrob Chemother* **72**, 2764-2768, doi:10.1093/jac/dkx217 (2017).

5 Gupta, S. K. *et al.* ARG-ANNOT, a new bioinformatic tool to discover antibiotic resistance genes in bacterial genomes. *Antimicrob Agents Chemother* **58**, 212-220, doi:10.1128/AAC.01310-13 (2014).

6 Doster, E. *et al.* MEGARes 2.0: a database for classification of antimicrobial drug, biocide and metal resistance determinants in metagenomic sequence data. *Nucleic Acids Res* **48**, D561-D569, doi:10.1093/nar/gkz1010 (2020).

7 Pal, C., Bengtsson-Palme, J., Rensing, C., Kristiansson, E. & Larsson, D. G. BacMet: antibacterial biocide and metal resistance genes database. *Nucleic Acids Res* **42**, D737-743, doi:10.1093/nar/gkt1252 (2014).

8 Liu, B. & Pop, M. ARDB--Antibiotic Resistance Genes Database. *Nucleic Acids Res* **37**, D443-447, doi:10.1093/nar/gkn656 (2009).

9 Gibson, M. K., Forsberg, K. J. & Dantas, G. Improved annotation of antibiotic resistance determinants reveals microbial resistomes cluster by ecology. *ISME J* **9**, 207-216, doi:10.1038/ismej.2014.106 (2015).

10 Saha, S. B., Uttam, V. & Verma, V. u-CARE: user-friendly Comprehensive Antibiotic resistance Repository of Escherichia coli. *J Clin Pathol* **68**, 648-651, doi:10.1136/jclinpath-2015-202927 (2015).

11 Naas, T. *et al.* Beta-lactamase database (BLDB) - structure and function. *J Enzyme Inhib Med Chem* **32**, 917-919, doi:10.1080/14756366.2017.1344235 (2017).

12 Srivastava, A., Singhal, N., Goel, M., Virdi, J. S. & Kumar, M. CBMAR: a comprehensive beta-lactamase molecular annotation resource. *Database (Oxford)* **2014**, bau111, doi:10.1093/database/bau111 (2014).

13 Gillespie, J. J. *et al.* PATRIC: the comprehensive bacterial bioinformatics resource with a focus on human pathogenic species. *Infect Immun* **79**, 4286-4298, doi:10.1128/IAI.00207-11 (2011).

14 Phelan, J. E. *et al.* Integrating informatics tools and portable sequencing technology for rapid detection of resistance to anti-tuberculous drugs. *Genome Med* **11**, 41, doi:10.1186/s13073-019-0650-x (2019).

15 Ezewudo, M. *et al.* Integrating standardized whole genome sequence analysis with a global Mycobacterium tuberculosis antibiotic resistance knowledgebase. *Sci Rep* **8**, 15382, doi:10.1038/s41598-018-33731-1 (2018).

16 Sandgren, A. *et al.* Tuberculosis drug resistance mutation database. *PLoS Med* **6**, e2, doi:10.1371/journal.pmed.1000002 (2009).

17 Flandrois, J. P., Lina, G. & Dumitrescu, O. MUBII-TB-DB: a database of mutations associated with antibiotic resistance in Mycobacterium tuberculosis. *BMC Bioinformatics* **15**, 107, doi:10.1186/1471-2105-15-107 (2014).
